# Efficacy predictions for omalizumab treatment based on basophil CD203c expression in patients with allergic rhinitis by basophil activation test -- a real-life, pilot study^☆^

**DOI:** 10.1016/j.waojou.2026.101388

**Published:** 2026-04-20

**Authors:** Zhan Zhao, Huidong Chen, Tianyi Liu, Jun Hong, Yuqin Deng, Qingquan Hua

**Affiliations:** a Department of Otolaryngology-Head and Neck Surgery, Renmin Hospital of Wuhan University, Wuhan, 430060, China; b Department of Otolaryngology, Peking Union Medical College Hospital, Research Units of New Technologies of Endoscopic Surgery in Skull Base Tumor (2018RU003), Chinese Academy of Medical Sciences and Peking Union Medical College, Beijing, 100730, China; c Department of Clinical Laboratory, Renmin Hospital of Wuhan University, Wuhan, 430060, China

**Keywords:** Basophil activation test, Omalizumab, CD63, CD203c, Allergic rhinitis

## Abstract

**Background:**

Basophils are key targets in allergic diseases. Omalizumab blocks IgE binding to FcεRI on basophils, reducing allergic responses. The basophil activation test aids allergy diagnosis, but their role in allergic rhinitis (AR) remains unclear. We assessed omalizumab's impact on basophil reactivity and its clinical associations in moderate-to-severe AR.

**Objective:**

This study aimed to determine the predictive value of CD203c and CD63 as basophil activation markers for clinical response to omalizumab in patients with moderate-to-severe allergic rhinitis.

**Methods:**

We collected and analyzed clinical symptoms and the Rhinoconjunctivitis Quality of Life Questionnaire (RQLQ) scores of 27 patients with moderate-to-severe AR who were treated with omalizumab for more than 12 weeks. Patients were divided into a non-basopenic group (OMA-AR/NB) and a basopenic group (OMA-AR/B) based on peripheral blood basophil counts (basopenic: <8000 cells/mL). FcεRI, CD63, and CD203c expression on blood basophils induced by stimulant were measured at baseline and at 12 weeks after omalizumab treatment using flow cytometry.

**Results:**

The RQLQ score in OMA-AR/NB decreased from 33.0 ± 11.0 at baseline to 5.2 ± 4.2 at 12 weeks, while in OMA-AR/B, the score decreased from 33.8 ± 7.6 at baseline to 19.4 ± 5.9 at 12 weeks. Additionally, the basophil activation assay yielded the best classification accuracy (73.97% sensitivity, 88.89% specificity, cut-off value: 8.0%) for %CD203c+ in patients with AR.

**Conclusion:**

During omalizumab treatment in patients with AR, CD203c was a more suitable marker for the basophil activation test (BAT). Furthermore, %CD203c^+^ > 8.0%, combined with a normal basophil count at baseline, could be used to predict the therapeutic effect of omalizumab.

## Graphical abstract

During omalizumab treatment for allergic rhinitis, %CD203c^+^ is superior to %CD63^+^ as a monitoring index in basophil activation test. %CD203c^+^ decreased more significantly and the symptoms of allergic rhinitis improved more rapidly after OMA treatment in OMA-AR/NB. On the contrary, %CD63^+^ decreased more significantly and the symptoms of allergic rhinitis eased more slowly after OMA treatment in OMA-AR/B. AR, allergic rhinitis; B, basopenic; BAT, basophil activation test; %CD203c^+^, the ratio of CD203c^+^ basophils in BAT; %CD63^+^, the ratio of CD63^+^ basophils in BAT; HC, healthy control; NB, non-basopenic; OMA, omalizumab; ROC, receiver operating characteristics; RQLQ, Rhinoconjunctivitis Quality of Life Questionnaire; V1/2/3/4, study visit. The figure was drawn by Figdraw (www.figdraw.com).

**Figure:**
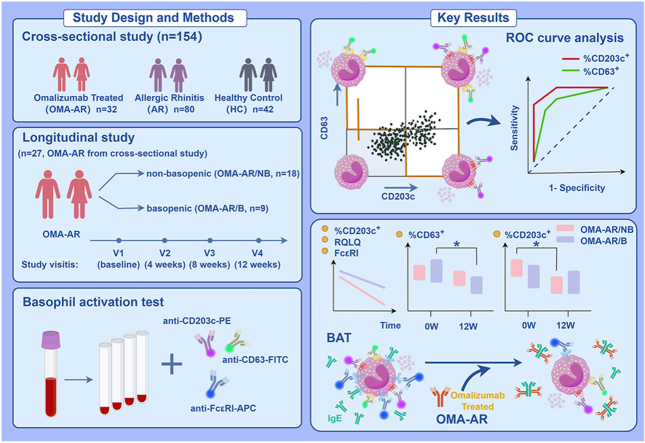

## Introduction

Allergic rhinitis (AR) is one of the most common diseases worldwide, affecting 10%–40% of the global population and causing a considerable burden on both rhinitis sufferers and society.^1^ The prevalence of AR has increased markedly in many countries over the past 30 years, with a notable rise in moderate-to-severe cases.^2^ The characteristic clinical manifestations of AR include nasal itching, sneezing, rhinorrhea, and nasal congestion. Ocular symptoms, such as red eyes, itchy eyes, and tearing, are often present, indicating the common co-occurrence of allergic rhinoconjunctivitis (AR/C).^3^ The economic burden and associated costs of AR are substantial.^4^ AR is a symptomatic nasal disorder caused by an immunoglobulin E (IgE) mediated inflammation of the nasal mucosa following allergen exposure.^5^ The management of AR primarily involves allergen avoidance and environmental controls. Symptomatic relief is achieved with conventional pharmacotherapy (eg, antihistamines, corticosteroids), whereas allergen immunotherapy (AIT) is a disease-modifying treatment aimed at inducing immunologic tolerance.^6^ However, many patients with moderate-to-severe AR remain dissatisfied with the efficacy of traditional medications. Conventional therapies provide only temporary symptomatic relief, and some patients are prone to recurrent symptoms, which seriously affect the daily lives of patients with moderate-to-severe AR.^7^, ^8^, ^9^

Omalizumab, a humanized monoclonal antibody against IgE, is the first IgE-targeted therapy. It specifically recognizes and binds to IgE antibodies in serum, preventing IgE from binding to high-affinity IgE receptors (FcεRI) on mast cells and basophils.^10^^,^^11^ This mechanism lowers free IgE levels in the circulation and interrupts the allergic cascade.^12^ Omalizumab was first approved for use in Australia in 2002, initially for severe uncontrolled asthma in adults.^13^ Subsequently, its indications have expanded, and it is now approved by the U.S. Food and Drug Administration (FDA) for chronic spontaneous urticaria (CSU), food allergy, and severe allergic asthma.^14^, ^15^, ^16^ Several studies have demonstrated that omalizumab reduces serum free IgE levels and provides clinical benefit in patients with AR.^17^^,^^18^ These benefits are related to a reduction in FcεRI expression and function, as well as anti-inflammatory effects on cell surface markers in blood and nasal tissue 19, 20, 21. Omalizumab also rapidly alleviates all nasal symptoms and improves scores on the Rhinoconjunctivitis Quality of Life Questionnaire (RQLQ).^22^ As a biological agent, omalizumab cannot achieve a cure of AR, its main role is to control the symptoms in patients. Furthermore, some patients with AR do not respond well to this anti-IgE therapy. The prediction of clinical responses to omalizumab in patients with AR is not particularly clear and still needs further investigation.^23^ To help patients, it is crucial to evaluate the therapeutic effect of omalizumab at an early stage and select the most appropriate treatment plan for achieving the best therapeutic outcome. Therefore, we attempted to identify a marker that could predict and monitor the efficacy of omalizumab.

The basophil activation test (BAT) utilizes basophil activation status as a biomarker to detect allergic diseases. In BAT, activation is indicated by an increased expression of CD63 and/or CD203c on the surface of the basophils induced by antigen stimulation. CD63 serves as a precise membrane marker of anaphylactic degranulation and is barely detectable on the cell surface without antigen stimulation.^24^ CD203c, a glycosylated type II transmembrane protein, is constitutively present on the surface of unstimulated basophils, and its surface expression increases further upon activation. Activation of basophils can be detected through the upregulation of specific surface markers, with CD63 and CD203c being the most widely used activation marker.^25^^,^^26^ As an *in vitro* allergen test, BAT offers advantages such as high safety and specificity. It can be used as a part of *in vitro* diagnostic evaluation for patients with food, insect venom and drug allergies to identify clinically relevant allergens. Additionally, BAT has been used to monitor AIT in allergic patients, with studies highlighting its clinical value in monitoring AIT outcomes.^27^^,^^28^

In anti-IgE therapy, omalizumab binds serum free IgE, resulting in downregulation of FcεRI on the surface of basophils and a decrease in serum free IgE levels.^29^ However, studies have shown that there is no significant correlation between serum free IgE levels and clinical efficacy. Moreover, some studies have found that changes in FcεRI on basophils are related to the changes in clinical symptoms in CSU.^30^^,^^31^ However, the studies on the specific mechanisms underlying changes in FcεRI on the surface of basophils and the association between FcεRI changes and basophil reactivity are limited, particularly in the context of omalizumab treatment for AR. Therefore, our study analyzed the changes in basophil reactivity in patients with moderate-to-severe AR before and after omalizumab treatment, as well as its potential association with clinical response.

## Method

### Subjects and study design

#### Patients

**Fig. 1:**
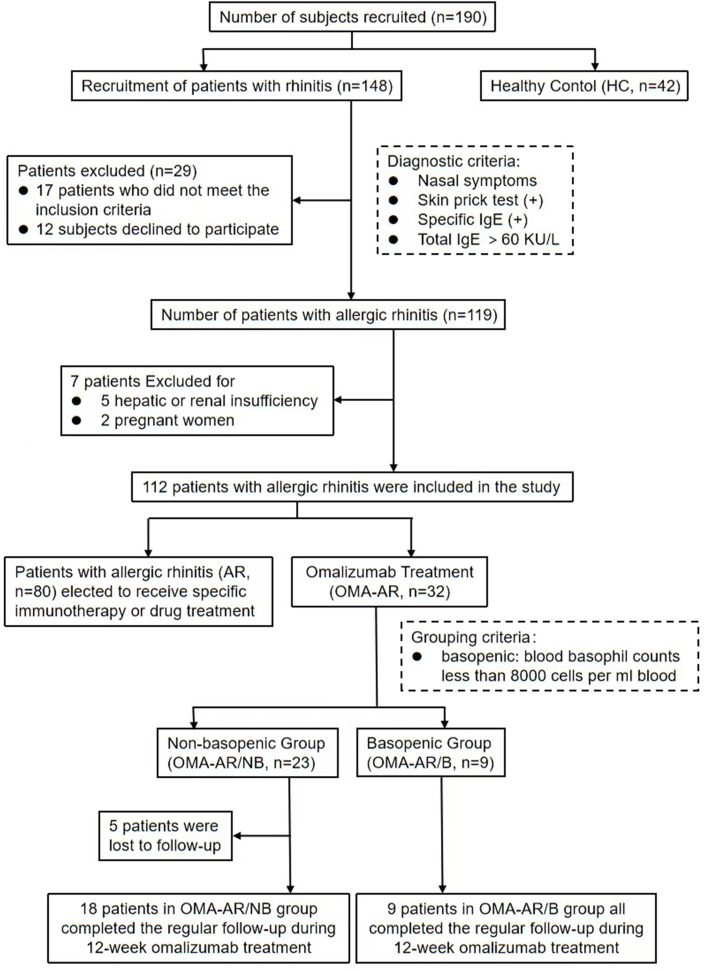
Study design.

In this retrospective cohort study, a total of 112 patients with uncontrolled moderate-to-severe AR and 42 healthy controls were enrolled between January 2024 and October 2024 from the ENT Allergic Reactions Clinic at Renmin Hospital of Wuhan University (Fig. 1). Among the 112 enrolled patients with AR, thirty-two patients received omalizumab treatment. And the study met statistical requirements based on the sample size calculation. All patients had a self-reported physician diagnosis of moderate-to-severe AR, and all allergic diagnoses were further substantiated by patient medical history, skin prick test (SPT), allergen-specific IgE (sIgE), and total IgE levels.

Patients were included in the study based on the following inclusion criteria: they satisfied the clinical diagnostic criteria outlined in the latest AR treatment guidelines;^32^ tested positive for allergens through either allergen-specific IgE or total serum IgE, and demonstrated a strong positive (**+++**) reaction in the allergen SPT; had total serum IgE levels between 60 KU/L and 3000 KU/L before treatment; serum specific IgE (sIgE) to Der p and/or Der f ≥ 0.7 kUA/L; were able to cooperate with the medical staff and complete relevant questionnaires regularly; and had a poor response to traditional drug treatment.

Patients were excluded from the study based on the following criteria: having hepatic or renal insufficiency; receiving other monoclonal antibody treatments; being allergic to the drug components of omalizumab; having parasitic infections, autoimmune diseases, immune deficiency diseases, or any other systemic disease that could elevate total IgE levels; having taken oral steroids for any condition within the prior 3 months; having total serum IgE levels outside the range of 60 KU/L to 3000 KU/L before treatment; being younger than 6 years old or older than 60 years old; or being pregnant.

### Clinical variables

Clinical variables were evaluated using the RQLQ and the Rhinitis Control Assessment Test (RCAT). Twenty-seven patients completed monthly subcutaneous injections of omalizumab for at least 12 weeks, with dosages determined based on the standard omalizumab dosage table. Clinic visits were conducted before each injection, and questionnaires were completed at the end of the 12-week treatment period.

### Basophil activation test (BAT)

For the BAT, 100 μL of heparinized blood was incubated with 100 μL of anti-IgE (BD, San Jose, CA,USA) for 15 min at 37 °C. The other tubes contained the fMLP positive control and the PBS negative control. Samples were then washed with PBS-EDTA (Neobioscience, Shenzhen, China) and labeled with CD45-PerCP, FcεRI-APC, CD63-FITC, and CD203c-PE (all purchased from Biolegend, San Diego, CA, USA). Erythrocytes were subsequently lysed with Lysing Buffer (BD, San Jose, CA,USA), washed with PBS-BSA (Cellmax, Lanzhou, China), and analyzed using a BD FACS Calibur flow cytometer. Basophils were identified using a sequential gating strategy. First, lymphocytes were gated based on forward and side scatter (FSC/SSC) properties. Within this lymphocyte gate, basophils were then identified as CD45^+^FcεRI^+^ cells. The activation status of these gated basophils was subsequently assessed by measuring the surface expression of CD63 and CD203c. Basophil reactivity can be measured using %CD63^+^ or %CD203c^+^ basophils. The mean fluorescence intensity (MFI) was measured to quantify the surface expression levels of basophil surface markers.

### Serum total and specific IgE

Total and specific IgE levels were measured using ImmunoCAPs (ThermoFisher Scientific).

### Statistical analysis

Categorical data (eg, sex) are presented as counts and percentages. Data with continuous distribution (eg, age) are expressed as mean ± standard deviation. Continuous data were compared with the two-sample t-tests or Kruskal–Wallis tests with multiple comparisons, and categorical data with Fisher's exact test. Mann–Whitney *U* test was used to analyze non-normally distributed data. SPSS 27.0 statistical software was used for all statistical analyses. Data analysis was performed using GraphPad Prism 9.5 software, which was used to calculate statistical significance (P < 0.05). The sample size was calculated using the standard formula for comparing means: n = [(Zα/2+Zβ)^2^ × σ^2^]/δ^2^.

## Results

### Participant characteristics

**Fig. 2:**
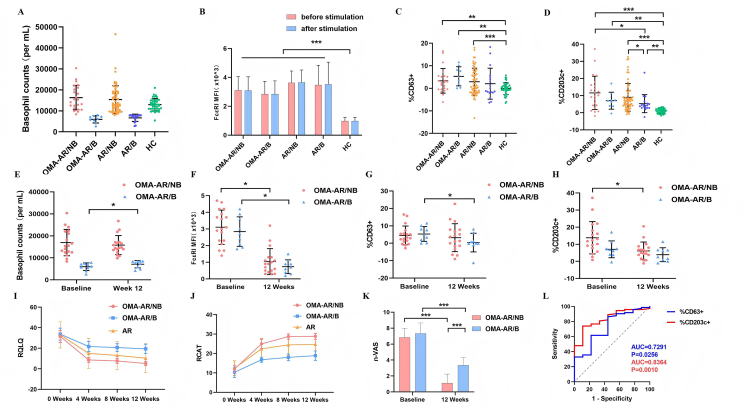
**A-D** Comparison of expression levels of basophilic granulocyte surface markers or basophil counts among all groups at baseline. **A,** the change of expression level of FcεRI before and after stimulation in BAT. **B,** basophil counts in each group at baseline. **C,D,** comparison of the baseline proportion of CD203+/CD63+ basophils in each group. **E-H** Comparison of expression levels of basophilic granulocyte surface markers or basophil counts between OMA-AR/NB and OMA-AR/B during omalizumab treatment. **I-K** Comparison of RQLQ, RACT and n-VAS between OMA-AR/NB and OMA-AR/B during omalizumab treatment. **L,** ROC Curve of %CD203^+^ and %CD63^+^.

A total of 112 patients with AR were enrolled, of which 32 chose to receive omalizumab treatment. Patients with AR who received omalizumab were divided into the basopenic group (OMA-AR/B, n = 9) and the non-basopenic group (OMA-AR/NB, n = 23) based on their whole blood basophil counts (basopenic: blood basophil counts <8000 cells/mL blood) 33, 34, 35. Among the 23 patients in the OMA-AR/NB group, five were lost to follow-up, leaving 18 patients who completed omalizumab treatment. All nine patients in the OMA-AR/B group completed the treatment. Patients with AR who received specific immunotherapy or drug treatment were divided into the basopenic group (AR/B, n = 18) and the non-basopenic group (AR/NB, n = 62). The baseline demographic and clinical characteristics of these subgroups are summarized in Table 1 and Table 2, while the corresponding baseline levels of basophil counts and surface markers are presented in Fig. 2 A-D.

**Table 1:** Demographic and baseline clinical characteristics of study participants. Values are mean ± standard deviation. a, two-sample *t*-test; b, Fisher exact test.

	OMA-AR (n = 32)	AR (n = 80)	HC (n = 42)	*p* value
Age (years)	28.3 ± 18.6	24.5 ± 15.2	25.0 ± 19.3	0.442
Male (n,%)	22 [69]	52 [65]	22 [52]	0.278
Body weight (kg)	55.0 ± 15.3	50.8 ± 16.2	44.4 ± 19.5	0.046
Smoker (n,%)	5 [16]	8 [10]	4 [10]	0.649
Family history (n,%)	21 [66]	54 [68]	7 [17]	<0.001
Asthma (n,%)	5 [16]	9 [11]	0	0.044
Urticaria (n,%)	3 [9]	5 [6]	0	0.166
Conjunctivitis (n,%)	3 [9]	7 [9]	0	0.135
Food allergy (n,%)	2 [6]	4 [5]	0	0.298
Inhaled corticosteroids (n,%)	7 [22]	14 [18]	0	0.009
Total serum IgE (KU/L)	491.1 ± 635.7	372.2 ± 402.0	11.0 ± 5.6	<0.001
Basohpil counts (/mL)	13,412 ± 6868	13,439 ± 6900	12,966 ± 2890	0.726

Abbreviations: RCAT, rhinitis control assessment test score; n-VAS, nasal-visual analog scale score of rhinitis symptoms; RQLQ, rhinoconjunctivitis quality-of-life questionnaire score

**Table 2:** Demographic and baseline clinical characteristics of OMA-AR. Values are median (interquartile range) or mean ± standard deviation.

	OMA-AR/NB(n = 23)	OMA-AR/B (n = 9)	*p* value
Age (years)	30.5 ± 20.5	22.7 ± 11.4	0.181
Male (n,%)	15 [65]	7 [78]	0.507
Body weight (kg)	55.0 ± 15.3	55.0 ± 16.2	1.000
Smoker (n,%)	4 [17]	1 [11]	0.672
Family history (n,%)	16 [70]	5 [56]	0.469
Asthma (n,%)	4 [17]	1 [11]	0.672
Urticaria (n,%)	3 [13]	0	0.083
Conjunctivitis (n,%)	2 [9]	1 [11]	0.840
Food allergy (n,%)	1 [4]	1 [11]	0.493
Inhaled corticosteroids (n,%)	5 [22]	2 [22]	0.977
D.Pteronyssinus-slgE (KU/L)	31.5 ± 32.0	21.4 ± 21.8	0.393
D.farinae-slgE (KU/L)	41.2 ± 34.0	33.3 ± 25.1	0.534
Foods mix-slgE (KU/L)	0.08 ± 0.19	0.18 ± 0.50	0.408
Weed pollen mix-slgE (KU/L)	0.05 ± 0.14	0.11 ± 0.32	0.475
Total serum IgE(KU/L)	443.7 ± 511.4	612.3 ± 907.6	0.509
Omalizumab dose (mg/month)	150 (150–300)	150 (150–300)	0.869
Basohpil counts (/mL)	16,322 ± 5820	5973 ± 1735	<0.001
RQLQ	33.0 ± 11.0	33.8 ± 7.6	0.848
RCAT	11.3 ± 3.8	10.3 ± 2.6	0.491
n-VAS	7.0 ± 1.2	7.3 ± 1.3	0.552

Abbreviations: RCAT, rhinitis control assessment test score; n-VAS, nasal-visual analog scale score of rhinitis symptoms; RQLQ, rhinoconjunctivitis quality-of-life questionnaire score

### Changes in symptom response during omalizumab treatment

RCAT scores increased in both the OMA-AR/NB and OMA-AR/B groups during omalizumab treatment, but the increase in OMA-AR/NB was more rapid than that in OMA-AR/B. In OMA-AR/NB group, RCAT scores increased from 11.3 ± 3.8 at baseline to 28.8 ± 3.0 at 12 weeks (P < 0.001), and the RCAT score in OMA-AR/B increased from 10.3 ± 2.6 at baseline to 18.9 ± 2.4 at 12 weeks (P < 0.001). During omalizumab treatment, the RQLQ score decreased in both groups, with the OMA-AR/NB group experiencing more rapid decline than the OMA-AR/B group. The RQLQ score in OMA-AR/NB decreased from 33.0 ± 11.0 at baseline to 5.2 ± 4.2 at 12 weeks (P < 0.001), while the RQLQ score in OMA-AR/B decreased from 33.8 ± 7.6 at baseline to 19.4 ± 5.9 at 12 weeks (P < 0.001) (Fig. 2. I-K; Table 3).

**Table 3:** Demographic and clinical characteristics of OMA-AR during omalizumab treatment. Values are mean ± standard deviation. ∗∗∗P < 0.001.

	Baseline (n = 32)			12 Weeks (n = 27)		
	OMA-AR/NB	OMA-AR/B	*p* value	OMA-AR/NB	OMA-AR/B	*p* value
	(n = 23)	(n = 9)		(n = 18)	(n = 9)	
Total serum IgE(KU/L)	443.7 ± 511.4	612.3 ± 907.6	0.509	870.7 ± 728.3	1021.4 ± 1486.9	0.724
%CD63^+^	3.3 ± 5.5	5.3 ± 4.2	0.332	3.2 ± 8.0	0.4 ± 5.3	0.355
%CD203c^+^	11.6 ± 9.7	7.1 ± 4.9	0.195	6.2 ± 5.1	3.9 ± 4.1	0.257
FcεRI MFI (x10ˆ^3^)	3.1 ± 1.0	2.8 ± 0.9	0.492	1.0 ± 0.8	0.7 ± 0.4	0.284
Basohpil counts (/mL)	16,322 ± 5820	5973 ± 1735∗∗∗	<0.001	15,806 ± 4332	7125 ± 1674∗∗∗	<0.001
RQLQ	33.0 ± 11.0	33.8 ± 7.6	0.848	5.2 ± 4.2	19.4 ± 5.9∗∗∗	<0.001
RCAT	11.3 ± 3.8	10.3 ± 2.6	0.491	28.8 ± 3.0	18.9 ± 2.4∗∗∗	<0.001
n-VAS	7.0 ± 1.2	7.3 ± 1.3	0.552	1.1 ± 1.1	3.3 ± 1.0∗∗∗	<0.001

Abbreviations: RCAT, rhinitis control assessment test score; n-VAS, nasal-visual analog scale score of rhinitis symptoms; RQLQ, rhinoconjunctivitis quality-of-life questionnaire score

### Changes in %CD63^+^ basophils before and after activation during omalizumab treatment

In OMA-AR/NB group, the change in %CD63^+^ basophils was not significant during omalizumab treatment (Fig. 2. G). However, in the OMA-AR/B group, the %CD63^+^basophils decreased from 5.3 ± 4.2 at baseline to 0.4 ± 5.3 at 12 weeks (P = 0.046). The decrease of %CD63^+^ basophils in OMA-AR/B group was more pronounced.

### Change in %CD203c^+^ basophils before and after activation during omalizumab treatment

The %CD203c^+^ basophils in OMA-AR at week 12 were significantly lower than baseline levels during omalizumab treatment (Fig. 2. H). In OMA-AR/NB group, the %CD203c^+^ decreased from 11.6 ± 9.7 at baseline to 3.2 ± 8.0 at 12 weeks (P < 0.001), whereas in the OMA-AR/B group, the %CD203c^+^ decreased from 7.1 ± 4.9 at baseline to 3.9 ± 4.1 at 12 weeks (P = 0.156). The decrease in %CD203c^+^ basophils was more pronounced in the OMA-AR/NB group, while the %CD203c^+^ basophils in OMA-AR/B were not significantly different before and after omalizumab treatment.

### Comparison of sensitivity of %CD63^+^ and %CD203c^+^ basophils in BAT

The clinical therapeutic effect of omalizumab was evaluated based on the RQLQ scores of patients in OMA-AR. Combined with changes in the %CD63^+^ and %CD203c^+^ basophils before and after omalizumab treatment, the sensitivity and specificity of CD63 and CD203c surface markers on basophils in the BAT were analyzed. In the ROC curve, the area under the curve for CD203c was 0.8364 (73.97% sensitivity and 88.89% specificity), while that for CD63 was 0.7291 (61.64% sensitivity and 77.78% specificity) (Fig. 2. L). This indicates that CD203c is a more suitable surface marker than CD63 for evaluating BAT. In addition, the cut-off value of CD203c is 8%.

### Changes in basophil counts during omalizumab treatment

In the AR-OMA/NB group, the number of basophils was 16,322 ± 5820 at baseline and 15,806 ± 4332 after 12 weeks of omalizumab treatment (P > 0.05). And there was no significant difference in this change. In the AR-OMA/B group, the number of basophils increased more significantly (Fig. 2. E), from 5973 ± 1735 at baseline to 7125 ± 1674 per mL at 12 weeks of omalizumab treatment (P < 0.001).

### Changes in the expression of FcεRI during omalizumab treatment

There was no significant difference in the mean fluorescence intensity (MFI) values of FcεRI on basophils before and after BAT (Fig. 2. A). However, the expression of FcεRI on the surface of basophils in patients with AR was significantly lower than that of basophils at baseline after 12 weeks of omalizumab treatment (Fig. 2. F). In the OMA-AR/NB group, the MFI values of FcεRI on the surface of basophils decreased from 3.1 ± 1.0 at baseline to 1.0 ± 0.8 after 12 weeks of treatment (P < 0.001). In the OMA-AR/B group, the MFI values of FcεRI on the surface of basophils decreased from 2.8 ± 0.9 at baseline to 0.7 ± 0.4 after 12 weeks of treatment (P < 0.001).

## Discussion

Omalizumab, a monoclonal anti-IgE antibody, improves clinical outcomes in allergic diseases by reducing free IgE and downregulating FcεRI on basophils.^36^^,^^37^ However, FcεRI downregulation alone does not consistently correlate with clinical response, suggesting that functional changes in basophils may be more relevant.^38^^,^^39^ To investigate this, we applied BAT to assess changes in basophil responsiveness before and after omalizumab treatment in patients with AR.

Basophils and mast cells are the primary effector cells in IgE-mediated (type I hypersensitivity) allergic reactions. Abnormal basophil function and basopenia are related to disease activity in CSU, as well as basophil recruitment in CSU skin lesions.^40^^,^^41^ Notwithstanding the distinct pathophysiology between CSU and allergic rhinitis (AR), the central role of basophils in type I hypersensitivity provided a rationale for investigating whether basophil numeration could serve as a *trans*-disease biomarker. In line with this, we observed in our AR cohort that peripheral basophil counts also correlated with symptom severity. Therefore, we grouped the patients with AR receiving omalizumab according to the basophil counts in peripheral blood. During activation and degranulation, basophils expressed unique surface markers such as CD63 and CD203c. The expression of CD203c on the surface of basophils decreased more significantly in AR-OMA/NB after 12 weeks of omalizumab treatment than in AR-OMA/B during BAT. Conversely, CD63 expression decreased more significantly in AR-OMA/B than in AR-OMA/NB during BAT (Fig. 2G). Combined with the clinician's assessment of patient clinical symptom improvement, CD203c was found to be a more suitable marker than CD63 for detecting basophils in omalizumab-treated patients with AR. We speculated that the significant differences in CD203c and CD63 expression in basophils of different individuals receiving the same stimulus may be related to the disease severity and the functional status of basophil. Therefore, changes in basophil function and counts in peripheral blood may be closely related to the progression of allergic diseases.

Basophils play a crucial role in allergic diseases, especially in CSU and other diseases. Low FcεRI reactivity of basophils can predict severe disease activity in CSU.^34^ In our study, the expression level of FcεRI on the surface of basophils in both AR-OMA/NB and AR-OMA/B decreased significantly after 12 weeks of omalizumab treatment. However, no significant difference was observed in the decrease of FcεRI expression level between the two groups. Clinically, a significant difference was observed in the decrease of RQLQ score between AR-OMA/B and AR-OMA/NB during omalizumab treatment. Therefore, the decrease in FcεRI expression could not predict the severity of AR in our study. We hypothesized that the reduction in FcεRI expression on basophil surface may not represent changes in basophil function. However, the studies on the changes in basophil function in AR are limited. Hence future research should focus on the changes in basophil function during the progression of AR disease.

**Fig. 3:**
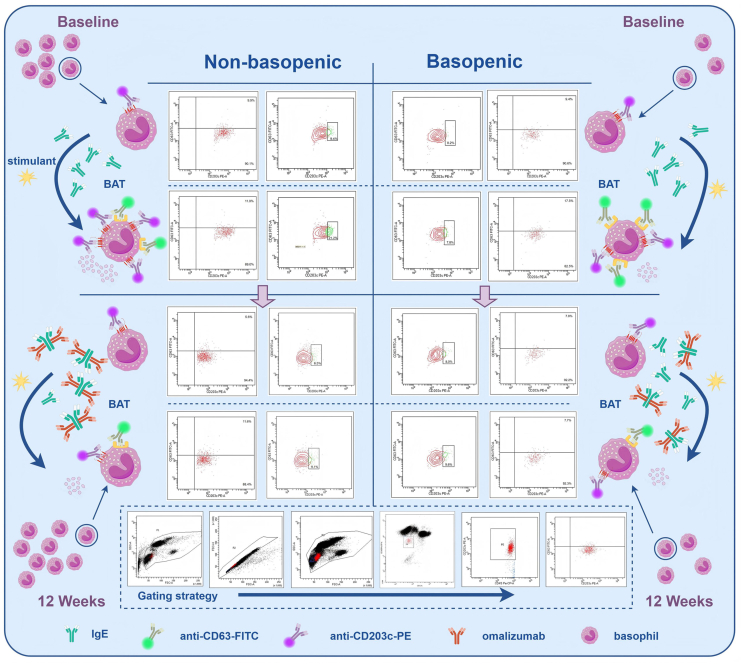
Basophils express various molecules which can be used to identify and measure their activation status by flow cytometry in basophil activation test (BAT). In this study, CD63 and CD203c were detected to evaluate the activation status of basophils. Blood basophil counts increase in all patients with AR after 12 weeks' omalizumab treatment. In OMA-AR/NB, both the increase of basophil counts and the decrease of %CD203c^+^ in BAT are more significant during OMA treatment. Basophil reactivity in OMA-AR/B is even lower at baseline, and the decrease of %CD63^+^ is more pronounced after 12 weeks of omalizumab treatment. The figure was drawn by Figdraw (www.figdraw.com).

**Fig. 4:**
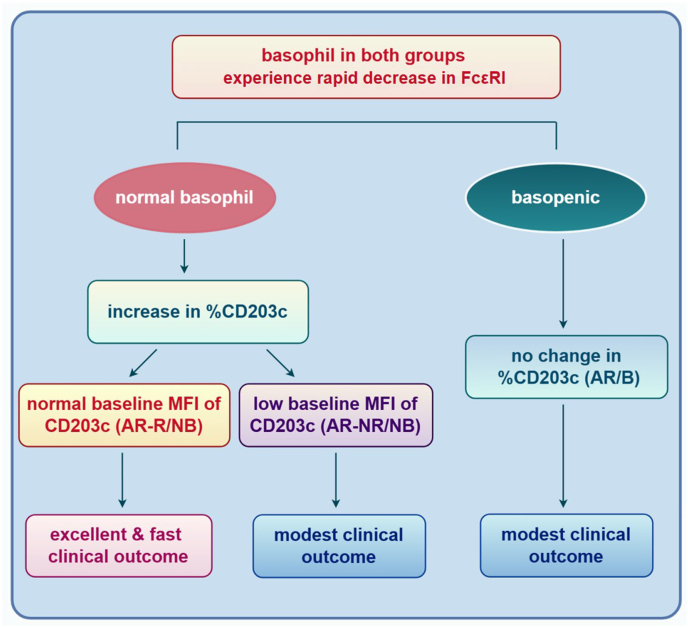
During omalizumab treatment, the relationship between basophil response in BAT and clinical efficacy in patients with AR. This study was based on the analysis of the experimental results of %CD203c^+^ and MFI of CD203c, and then divided according to the clinical responses of OMA-AR/NB and OMA-AR/B. This logical process is simplified to 2 steps and the expected clinical outcome. The figure was drawn by Figdraw (www.figdraw.com).

Although the decrease in the FcεRI expression during omalizumab treatment did not differ significantly between AR-OMA/B and AR-OMA/NB groups, a significant difference in %CD203c^+^ expression was observed between the two groups at the baseline. Additionally, the expression of %CD203c^+^ on the surface of basophils in BAT was lower in AR-OMA/B than that in AR-OMA/NB group, indicating reduced basophils reactivity in AR-OMA/B before omalizumab treatment. Moreover, at the baseline of omalizumab treatment, not all patients in AR-OMA/NB group had %CD203c^+^ on the surface of basophils in BAT higher than 8%. Higher baseline %CD203c^+^ may indicate a better response to omalizumab (Fig. 3). Moreover, we observed that the rate of decrease in %CD203c + basophils correlated with the extent of clinical improvement. A more rapid decline in this marker predicted a better and faster clinical response to omalizumab, while a slower decline was associated with a more moderate therapeutic effect. Based on the data analysis of this study, we predicted the clinical efficacy of omalizumab in treating AR and created a simple clinical efficacy prediction mechanism diagram for clinicians' reference (Fig. 4).

Our study had some limitations, including a small sample size across subgroups. Because we collected specimens from a single center, the number of patients with AR receiving omalizumab was limited, and a smaller proportion of patients with basopenia had AR compared to those with other allergic diseases. Additionally, BAT requires fresh anticoagulant blood within 4 h *in vitro*, making specimen collection difficult. All of this limited us in terms of performing more functional tests on basophils in patients with AR treated with omalizumab. Furthermore, our research on the mechanisms underlying differences in CD203c and CD63 expression in basophils is also very limited. We hope to provide new research ideas for subsequent studies on basophil function.

In patients with AR treated with omalizumab, not all showed good clinical outcomes, and some still experienced unsatisfactory symptom control. Therefore, in-depth study of basophil function changes in anti-IgE treatment of AR can help to understand the specific mechanism of omalizumab in treating AR, as well as the impact on basophil function changes, providing a new research direction for subsequent biological therapy in allergic diseases.

BAT is a reliable tool for *in vitro* detection of basophil activation status and is promising for diagnosing and monitoring allergic diseases.^42^ In this study, we found that a baseline %CD203c^+^ > 8.0% in BAT, combined with a normal basophil count, could predict the therapeutic effect of omalizumab in patients with AR. In the future, large-scale, multi-center, prospective studies across diverse populations and various type I hypersensitivity disorders are needed to validate these findings, establish a robust, data-driven threshold, and ultimately facilitate its translation into clinical practice. Meanwhile, we expect that in future clinical settings, %CD203c^+^ > 8% in BAT can help predict the clinical efficacy of omalizumab in patients with AR in advance.

## Abbreviations

allergic rhinitis (AR); Rhinoconjunctivitis Quality of Life Questionnaire (RQLQ); omalizumab-treated allergic rhinitis patients with normal basophil count (OMA-AR/NB); omalizumab-treated allergic rhinitis patients with basopenia (OMA-AR/B); immunoglobulin E (IgE); allergen immunotherapy (AIT); high-affinity IgE receptor (FcεRI); chronic spontaneous urticaria (CSU); basophil activation test (BAT).

## Authorship contribution

ZZ and HC are the co-first authors. ZZ collected the data, wrote original draft, performed data analysis, reviewed and edited. HC collected the data, performed data analysis, reviewed and edited. TL and JH collected the data. YD reviewed, edited, and supervised. QH conceived and designed, reviewed and edited, resourced, and supervised. All authors approved the final version of the manuscript to be published.

## Ethics approval

A total of 112 patients with uncontrolled moderate-to-severe AR and 42 healthy controls were enrolled between January 2024 and October 2024 from the ENT Allergic Reactions Clinic at Renmin Hospital of Wuhan University. All blood samples were collected with approval from the Ethics Committee of Renmin Hospital, Wuhan University. The need for written informed consent was waived by the Ethics Committee of Renmin Hospital, Wuhan University due to retrospective nature of the study (code number WDRY2024-K056).

## Declaration of Generative AI and AI-assisted technologies in the writing process

No generative AI or AI-assisted technologies were used in the creation of this work.

## Funding

This research was sponsored by the National Natural Science Foundation of China (82571280).

## Conflict of interest

No conflict of interest exits in this manuscript.
